# Bugs in the Bed: Addressing the Contradictions of Embedded Science with Agile Implementation Research

**DOI:** 10.9745/GHSP-D-20-00169

**Published:** 2021-03-31

**Authors:** James F. Phillips, Bruce B. MacLeod, S. Patrick Kachur

**Affiliations:** a Heilbrunn Department of Population and Family Health, Mailman School of Public Health, Columbia University, New York, NY, USA.; b Department of Computer Science, University of Southern Maine, Portland, ME, USA.

## Abstract

Implementation research often fails to have its intended impact on what programs actually do. Embedding research within target organizational systems represents an effective response to this problem. However, contradictions associated with the approach often prevent its application. We present case studies of the application of embedded implementation research in Bangladesh, Ghana, and Tanzania where initiatives to strengthen community-based health systems were conducted using the embedded science model. In 2 of the cases, implementation research standards that are typically embraced without question were abandoned to ensure pursuit of embedded science. In the third example, statistical rigor was sustained, but this feature of the design was inconsistent with embedded science. In general, rigorous statistical designs employ units of observation that are inconsistent with organizational units that managers can control. Structural contradictions impede host institution ownership of research processes and utilization of results. Moreover, principles of scientific protocol leadership are inconsistent with managerial leadership. These and other embedded implementation science attributes are reviewed together with contradictions that challenged their pursuit in each case. Based on strategies that were effectively applied to offsetting challenges, a process of merging research with management is proposed that is derived from computer science. Known as “agile science,” this paradigm combines scientific rigor with management decision making. This agile embedded research approach is designed to sustain scientific rigor while optimizing the integration of learning into managerial decision making.

## Abstract (precis)

Implementation research often fails to have its intended impact on what programs actually do. Embedding research within target organizational systems is an effective response to this problem. We present case examples from Bangladesh, Ghana, and Tanzania that demonstrate challenges associated with embedded science. We propose “agile science” as a means of sustaining scientific rigor while simultaneously catalyzing evidence utilization.

## Teaser

**Key Findings:**

- Embedding research into host program management and policy processes fostered the utilization of evidence for community-based health service development in Bangladesh, Ghana, and Tanzania. However, in each of these cases, strategies were required to offset challenges to the application of the embedded science approach.
- Research designs that maximize statistical rigor can be inconsistent with optimizing utilization. Achieving evidence-driven organizational change required phases for sequential learning to support systems development decision making.

**Key Implications:**

- Agile science methods can address the inherent contradictions of embedded science health systems development in low- and middle-income country settings without compromising scientific rigor.
- Research aims should target the goal of achieving programmatic improvement as an endpoint that supplements demographic and health goals. To that end, phases of agile implementation research can overlap and curate knowledge, facilitating the institutionalization of evidence-based learning.
- The principles of agile development are potentially relevant to health systems strengthening in low- and middle-income country settings.

## INTRODUCTION

Implementation science is often applied to developing health policies.^1^^–^^3^ In a recent review of the expanding application of this paradigm, Peters et al.^4^ defined implementation research as:

*… the scientific inquiry into questions concerning implementation—the act of carrying an intention into effect, which in health research can be policies, programmes, or individual practices (collectively called interventions).*

As they noted, research utilization is critical to ensuring that implementation science results have their intended impact. Various strategies have been advanced to foster implementation research utilization, such as “action research,”^5^^,^^6^ “participatory planning,”^7^ “applied research,”^8^ “operations research,”^9^ and “organization development.”^10^ Common to this body of literature are recommendations for establishing host organization ownership of research processes and outcomes.^11^^–^^13^ This focus on optimizing strategic ownership is termed “embedded science,”^14^^–^^17^ an implementation science strategy with a long history of development.^4^ Spanning several decades of methodological refinement,^18^ the study of research utilization has consistently shown that evidence-based decision making is enhanced by establishing a partnership of policy makers, implementers, and researchers in the design, conduct, interpretation, and dissemination of health systems research.^17^^,^^19^^–^^21^

This article reviews programs from Bangladesh, Ghana, and Tanzania that applied embedded implementation science to community-based primary health care systems development. Case studies identify challenges constraining the use of embedded science and strategies that each case employed to offset their effects on the integrity of implementation research results and utilization. The contradictions and challenges experienced in these case examples have also constrained the pursuit of embedded science elsewhere.^22^^–^^24^ All 3 case examples concerned the need for evidence-driven national community-based primary health care programs.^25^ Each case involved convening a partnership of a research team with a public program to configure embedded research designs, results, and procedures for translating results into action.

Although lessons emerge from the case studies, gaps in resolving contradictions are evident. We have identified a body of theory and action that has addressed these contradictions and could extend embedded implementation science in ways that could bridge these gaps. Developed by computer engineers and organizational scientists to simultaneously optimize systems performance and compliance with client needs, this application of implementation research is termed “agile science” ^26^:

*… a specific approach to project management for developing products rapidly and iteratively. Teams using an agile approach use principles and tools that involve inputs from end users, iterations on an idea, and frequent structured communication among all team members. Work is delivered more quickly with more appeal to end users and, ultimately, more value.*

We have identified a body of theory and action that addresses the gaps in resolving contradictions and could extend embedded implementation science to bridge these gaps.

We extract lessons from our case studies that resolve contradictions of embedded science, supplement these lessons with tenets of agile science that expand upon these lessons, and posit a synthesis of case lessons with agile implementation science that could address the full range of challenges that the 3 case examples portray.

## THREE EXAMPLES OF EMBEDDED RESEARCH SCIENCE

### Bangladesh: Evidence-Guided Community-Based Primary Health Care Development

The Matlab field station of the International Centre for Diarrhoeal Disease Research, Bangladesh (icddr,b) was founded in 1964 to provide a demographic platform for testing vaccines against cholera.^27^ Pursuing this goal required detailed information on a large population and longitudinal records of births, deaths, and migration.^28^ Termed a “demographic surveillance system” (DSS), this capability not only provided a basis for randomized trials of vaccine efficacy, but also permitted investigation of a wide range of sociodemographic topics, as well as the conduct of quasi-experimental studies of health systems issues.^29^

In the 1970s, such investigations were urgently needed. Having recently experienced a catastrophic typhoon followed by a devastating war of liberation and subsequently one of the most severe famines in the recorded history of South Asia, Bangladesh was widely regarded as a case example of the development challenges confronting the world's most impoverished settings. Matlab not only hosted a large population that exemplified the pervasive poverty and adversity of deltaic Bangladesh, its population dynamics were being continuously monitored by the DSS.

Bangladesh was particularly prominent in the global debate on population policy. Some commentators advanced the view that family planning service systems could not succeed in such a setting without prior development progress that would alter the demand for children.^30^ A contrasting perspective was advanced by health scientists who advocated the utilization of family planning programs as a core strategy of development assistance,^31^ a perspective derived from survey evidence that respondents who were not using contraception often stated that they wanted to space or limit childbearing. This finding suggested that “latent demand” for contraception was widespread even in settings where development challenges were pronounced. Matlab was deemed to be an ideal setting for testing the latent demand hypothesis by evaluating a project that would provide convenient access to modern contraceptive methods.

A 2-celled plausibility trial, the Contraceptive Distribution Project (CDP), was launched in 1977. Oral contraceptives and condoms were made freely available by traditional midwives who were trained to dispense supplies to women in their homes. Although initial results provided evidence of demand for contraception,^32^ adoption and continuation rates declined with time.^32^ The CDP had no lasting fertility impact.^33^

A series of qualitative appraisals were conducted during the CDP that informed the design of a follow-on study, the Family Planning Health Services Project (FPHSP). In response to community comments, the service provision role of traditional birth attendants was replaced with young literate women, called family welfare assistants (FWAs), who were trained and equipped to provide a wider range of contraceptive methods, treat common childhood illnesses, and purvey comprehensive childhood immunization services.^34^ FWAs provided fortnightly rounds of clinical and community outreach with backstopping at subdistrict health centers.

The total fertility rate of 6.8 births declined by nearly 2 births in the initial 18 months of FPHSP operation, and a rapid decline in childhood mortality followed.^33^^,^^35^^,^^36^ Matlab research was embedded in government operations to the extent that its design was developed collaboratively with the Planning Commission. However, its management, supervision, and monitoring omitted any discernable provision for governance by Ministry of Health and Social Welfare (MOHSW) officials (Box 1). This strategic gap was nonetheless essential to achieving the flexibility needed for developing and testing operational innovation.

BOX 1 **Does Success Always Matter?**

**Figure:**
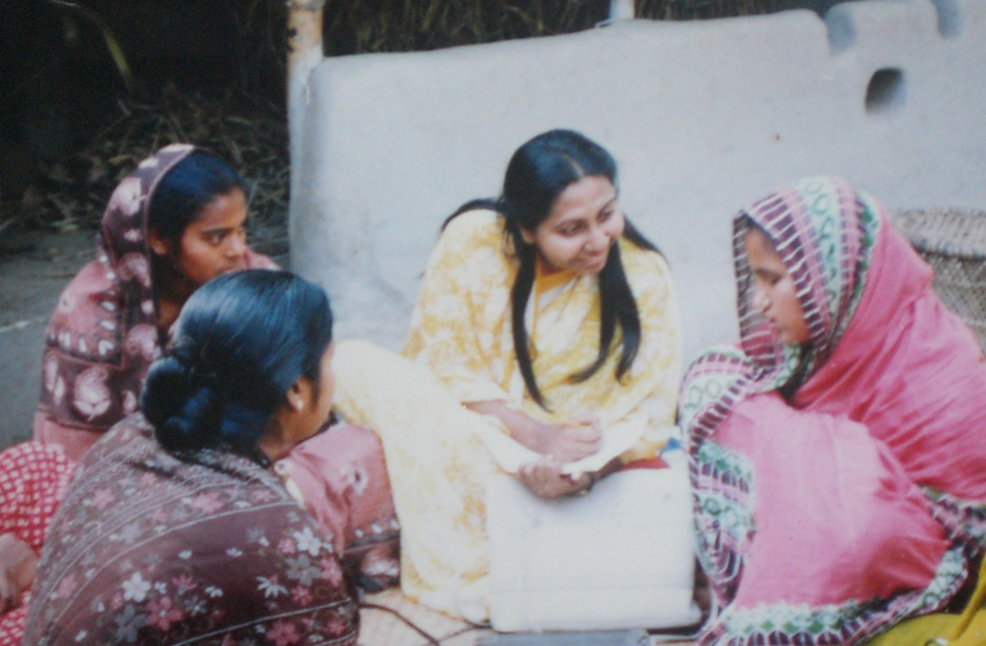
Community health service outreach session in Ghana with a Matlab family welfare assistant. © 1981 James F. Phillips/Population CouncilThe initial official reaction to the Matlab Family Planning Health Services Project results was decidedly negative, despite its impressive statistical and demographic evidence of success. In the view of stakeholders, statistical results provided no evidence that its operational staffing design and management system could be replicated by the Bangladesh Government program. To the official audience for Matlab results, its scientific results initially mattered less than the Maternal-Child Health-Family Planning Extension Project demonstration of the feasibility of replicating its strategies.

Matlab research was embedded in government operations; however, its management, supervision, and monitoring omitted any provision for governance by Ministry of Health and Social Welfare officials.

In 1982, the donor for this initiative, the United Nations Population Fund (UNFPA), sponsored a “tripartite review” of policy implications of these results. Participation included all institutions responsible for Matlab research, service, or policy development roles in maternal and child health. Despite their appreciation of Matlab results, senior MOHSW officials initially rejected the relevance of Matlab results to national programming because of fundamental staffing differences distinguishing Matlab from the national program. FPHSP frontline workers were icddr,b project employed FWAs, and existing MOHSW frontline workers were all male family welfare workers (FWWs). The FWWs had been deployed in the Pakistan era as smallpox eradication campaign workers and “encadered” into the civil service by the postliberation Bangladesh government as a large frontline primary health care workforce. In the view of MOHSW officials, civil service rules prevented any action that would replace FWWs.

Diplomacy prevailed. Tripartite review discussions successfully identified 7 themes that would guide future collaboration. First, the Maternal and Child Health–Family Planning Extension Project would test the transfer of Matlab service strategies to MOHSW district operations elsewhere in the country. Second, FWWs would be retained as “encadered” civil servants and deployed as health promotional workers. However, FWAs would be added to the system as temporary project employees of the MOHSW system and deployed with job descriptions and supervisory arrangements that were modeled on FPHSP operations. Third, the geographic density of FWA deployment would be consistent with MOHSW resources. The salaries of FWAs would be commensurate with government compensation scales. Fourth, all implementation activities would be MOHSW supervised, while all research components would be conducted by the icddr,b.^37^ Fifth, the extension project would be located in more than one district, with sites to be selected by the MOHSW. Frequent operational process reports would be channeled directly to MOHSW and the Planning Commission. Sixth, Matlab FPHSP operations would be sustained to permit research plans to be completed. Seventh, Matlab supervisors would serve as temporary advisors to MOHSW counterparts, but ongoing operations would be managed and supervised by MOHSW staff.

Early extension project results replicated the FPHSP impact. Contraceptive use in extension districts increased dramatically,^38^ and health service indicators improved as well. But most importantly, implementation demonstrated the feasibility of scaling up operations and process documentation was embedded in the MOHSW program monitoring operations.^39^ Because start-up activities coincided with national 5-year plan development processes, extension project field activities, costing data, and performance reports provided lessons that could be inserted into the strategic design of the World Bank's Third Health and Population lending agreement.^38^ This agreement, in turn, financed the hiring, training, and national deployment of 28,000 FWAs over the World Bank's Third Project 5-year implementation cycle. In this manner, extension project process experience was embedded into national program implementation plans well before final demographic results were attained.^40^^–^^42^ The program that emerged was inefficient,^43^ with staffing that included the continuing deployment of large numbers of unproductive FWWs. Yet it worked. The program was associated with dramatic improvements in reproductive and child health indicators and the onset of one of the most rapid demographic transitions ever recorded.^43^^–^^45^

The Extension Project process experience was embedded into national program implementation plans well before final demographic results were attained.

### The Ghana Community-Based Health Planning and Services Initiative

Ghana embraced the 1978 Alma Ata Declaration with policies that aimed to develop a community-based worker cadre. However, economic and political turmoil in the 1980s severely constrained progress. By 1990, nearly 2,000 “community health nurses” had been hired by the Ministry of Health (MOH) and trained to provide community-based primary health care. However, these nurses had been posted to inaccessible subdistrict clinics and hospitals because funds were unavailable for the construction of community health posts. Consequently, evidence compiled in the early 1990s consistently showed that “health for all by the year 2000” was unachievable unless reform was instituted.^46^ In response, MOH extended a mandate to the Navrongo Health Research Centre (NHRC), in Kassena-Nankana District of the Upper East Region (UER), to develop and test means of implementing community-based primary health care.^47^ The UER was known to have high mortality, prevalent morbidity of nutritional and infectious diseases,^48^^,^^49^ and other health development challenges.^50^

In 1992, a 6-week exchange visit to Bangladesh was convened for senior MOH officials and NHRC scientists^51^ to work with Matlab counterparts on the design of a DSS for evaluating research and a protocol for testing the transfer of the Matlab FPHSP service model to Ghana.^47^ General strategies of the Bangladesh program were potentially relevant to Ghana. However, operational details were not wholly transferable owing to profoundly contrasting cultural and organizational contexts of Ghana and Bangladesh (Box 2). Nonetheless, the Bangladesh phased progression of research was relevant to developing Ghana's community-based primary health care program. From its onset, community-based primary health care development in Ghana was a planned process of guiding organizational change with implementation research. A 3-community, 18-month participatory pilot phase was followed by a 4-celled district-wide plausibility trial that tested the fertility and mortality impact of alternative strategies for providing community-based primary health care.^47^

BOX 2 **What Was Transferable From Bangladesh to Ghana?**

**Figure:**
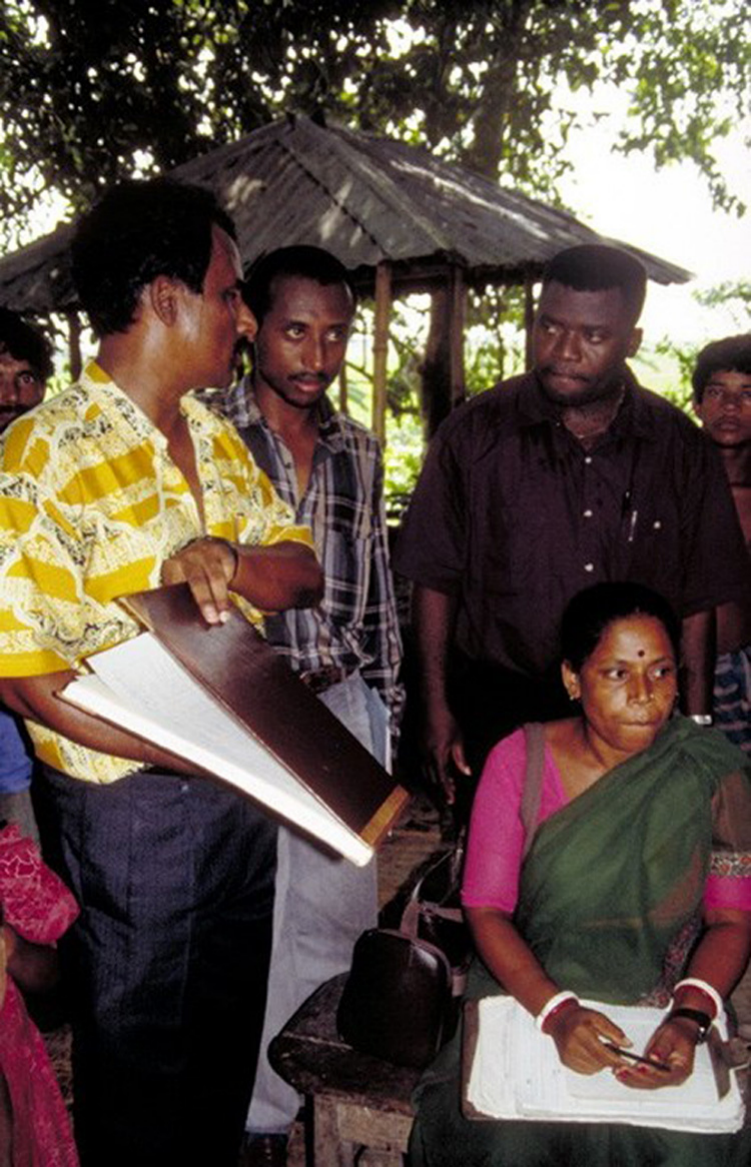
International Centre for Diarrheal Disease scientists in Bangladesh hosting Navrongo Health Research Centre counterparts in Matlab. © 1992 James F. Phillips/Population CouncilA 1993 exchange between the Navrongo service implementation and research teams and Matlab counterparts aimed to transfer the Matlab system to northern Ghana. Since social institutions, the health systems context, and the resource base in Navrongo were fundamentally different from the Matlab context, transferring the strategic details of the Matlab program to Africa did not make sense. What was transferable, however, was the process of developing a program that would work. In both examples, a diagnostic phase was followed by a plausibility trial and then by replication research. In both settings, this phased approach to systems development set the stage for scaling up evidence-based systems improvements.

When preliminary evidence emerging from Navrongo was promising, MOH convened a national health forum for deliberating on scaling up the Navrongo model. However, most participants were decidedly negative about taking this action because the NHRC resource base was believed to be nonreplicable and the cultural context atypical of other regions.

In the course of forum discussions, the Nkwanta District Director of Health Services advocated testing the transferability of the Navrongo system to his district where social characteristics contrasted markedly with the Navrongo setting. Within a year, a Nkwanta replication pilot generated promising implementation experience that was presented in a repeat forum. Based on consensus, MOH adopted the Navrongo service model and the Nkwanta replication strategy as a national policy known as Community-based Health Planning and Services (CHPS).^52^

CHPS implementation commenced in 2000. In the decade that followed, external donor support focused on expanding in-service and preservice training for frontline workers, promotional activities in donor-selected districts, and other topics of interest to donors. Imbalances ensued. This commitment to developing components of CHPS was implemented by contracting with international technical assistance agencies who engaged in operational management activities that bypassed the newly constituted Ghana Health Service (GHS). Budgetary support for bridging implementation gaps was peripheral to this type of commitment because international contracting agency technical assistance consumed most of the external revenue earmarked for CHPS. Most critically, no available mechanism paralleled the World Bank's sector-wide health and population lending that had enabled scale-up in Bangladesh. Lacking an overarching implementation plan with associated financing arrangements, CHPS implementation was decentralized to district management teams where its start-up financing depended upon volunteer labor and contributions from communities to be served. Lack of resources and the paucity of district leadership experience with community mobilization constrained the pace of CHPS scale-up during its first decade of operation. By 2008, GHS program monitors estimated that achieving total population coverage would require 49 years.

CHPS implementation was decentralized to district management teams; its start-up financing depended upon volunteer labor and contributions from communities to be served.

Workshops on donor-financed technical components of CHPS proliferated without implementation-based leadership training or financing for incremental start-up costs. Unlike Bangladesh, personnel availability was not a problem. The hiring and technical training of CHPS workers was expanded far more rapidly than MOH could expand community facilities and procure equipment that was essential for their deployment. Coverage achieved by 2008 was concentrated in the 32 districts where implementation teams had experienced on-site orientation visits in Nkwanta and had received start-up grants for launching CHPS in 1 or 2 demonstration communities.^53^ By establishing demonstration communities, equipped with seed revenue, district managers could undertake activities that catalyzed the diffusion of implementation within their districts. Although this approach was successful in participating districts, the Nkwanta CHPS development exchange strategy was terminated in 2004 because contracting mechanisms of donor agencies were inconsistent with the Nkwanta embedded research model.

In 2009, the MOH convened a qualitative appraisal to determine why some districts had implemented CHPS in nearly all communities, while most other districts had achieved hardly any progress at all.^54^ Leadership and financing were found to be the key factors. Resources for CHPS start-up costs were often available from district development financing mechanisms, but district health officials tended to rely upon internal GHS budgets rather than to explore means of seeking revenue elsewhere. When grassroots political leaders were properly engaged with CHPS community demonstration activities and were fully aware of its popularity, they tended to commit development revenue to community health post construction. Often this permitted community volunteer construction of interim facilities that could be used for CHPS until permanent facilities could be constructed. Investment in this approach enabled the program to commence without delay. Participants in Nkwanta exchanges understood the process of community engagement that generated this political support. With creative engagement of traditional leaders and grassroots politicians, managers were able to mobilize resources for rolling out CHPS sequentially, community by community, by linking leaders from communities that had not yet implemented CHPS with start-up activities in communities where CHPS milestones were progressing. This process of “guided diffusion” catalyzed spontaneous scaling up of CHPS operations from a few start-up communities to district-wide implementation.^55^

This organic process within districts contrasted with the World Bank financed “top-down” engagement of the health bureaucracy in Bangladesh. Scaling-up has eventually worked, covering all targeted communities in Ghana. As many studies of social diffusion have demonstrated, catalytic inputs are critical to getting started. Where diffusion was organized, with catalytic revenue provided for demonstration activities, district teams could implement CHPS. But, for the first decade of CHPS implementation, investment in the process and agile leadership for making it happen were typically lacking.

An organic process within districts in Ghana contrasted with the “top-down” financing in Bangladesh—scaling-up eventually worked, covering all targeted communities.

To develop and test mechanisms for reforming the CHPS implementation process, the GHS launched the Ghana Essential Health Interventions Program (GEHIP) in 2009 in 4 UER districts.^56^ Areas associated with prior NHRC research were excluded, while 7 other districts of the UER were comparison areas. In treatment districts, leadership exchanges were supplemented with flexible financing of US$0.85 per capita per year for 3 years. Existing leadership training was augmented with community-based participatory planning that included district health management team (DHMT) and district political and development personnel.

The impact of GEHIP on the pace of CHPS implementation was immediate, accelerating coverage from 20% of the target population to 100% in less than 4 years.^7^ Treatment district coverage at the end of GEHIP was achieved at twice the levels as in comparison districts. This coverage expansion was combined with development of an emergency referral and acute care system.^57^ Mortality declined in all UER districts, but GEHIP districts experienced a more pronounced mortality transition than comparison districts.^58^ GEHIP fertility effects were also evident.^59^

Evidence of GEHIP affordability and impact justified the adoption of its strategies as policy, and inspired an ongoing 5- year replication project in 2 other regions of Ghana, known as CHPS+.^53^^,^^60^ Designed to provide continuous knowledge of the replication process, CHPS+ has implemented 4 “system learning districts” where GEHIP capacity is fully functional and where visiting district implementation teams observe operations, learn from the process, and return to their home districts with small grants for financing the roll-out of lessons learned.

### The Tanzania Connect Project

In 2008, the President of Tanzania pledged to develop a “dispensary in every community” through a policy known by its Swahili acronym MMAM for Mpango wa Maendeleo wa Afya ya Msingi, translated as the Primary Health Services Development Program.^61^ This commitment coincided with the proliferation of community-based primary health care programs throughout Africa,^62^^–^^65^ often in response to international advocacy of the deployment of community health workers (CHWs). ^66^^–^^68^ But MMAM was controversial. At the time of the proclamation of MMAM, Tanzania had the highest geographic density of primary health care fixed facilities in Africa.^69^ The added value of adding several thousand CHWs was questionable in a setting where accessible fixed-facility services were already functioning. A project was developed to respond to the need for a trial of the MMAM policy, while also addressing international policy questions concerning the health and survival benefits of CHW deployment. This project, known as Connect, would develop procedures for MMAM implementation and test the health and survival impact of this policy.

Unlike district systems development interventions in Bangladesh and Ghana, Tanzanian policy questions concerned the value of adding community-level doorstep care in an existing community-based system of fixed-facility dispensary services. In this instance, research advisors to MOHSW believed that a randomized controlled trial of CHW deployment would be appropriate because randomization of community catchment areas was both organizationally and statistically feasible. Moreover, addressing policy questions concerning CHW effects with statistically rigorous randomized designs remained rare.^70^^,^^71^ Since Tanzania had a long legacy of public investment in community dispensary care, the existing system provided a platform for testing the proposition that CHW household outreach could have incremental benefits, even in the context of accessible fixed-facility care.

The Ifakara Health Institute (IHI) was an appropriate institution for undertaking this trial (Box 3). The IHI DSS had functioned for over a decade in households located in 101 communities of 3 rural districts.^72^ The DSS operations provided a statistical platform for testing the hypothesis that CHW deployment saves lives in the context of accessible dispensary-based care.^73^

BOX 3 **Rigor as a Barrier to Success**

**Figure:**
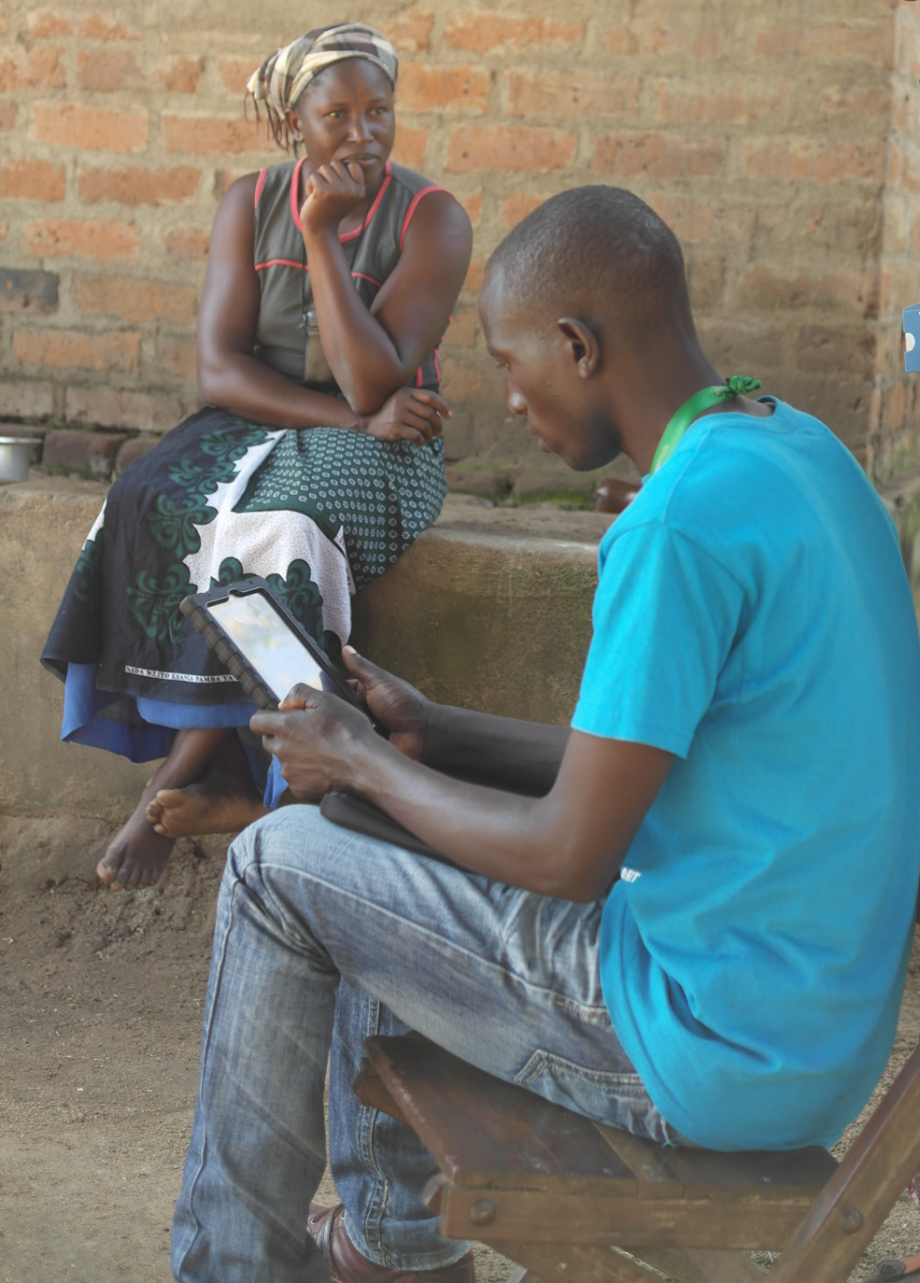
Ifakara Health Institute demographic surveillance system interview, Rufiji District, Tanzania. © 2012 James F. Phillips/Columbia UniversityThe Tanzania Connect experiment was a randomized controlled trial that conformed to conventional standards of statistical rigor. Its use of demographic surveillance permitted community randomization of the assignment of community health workers (CHWs). While this was statistically appropriate, the relevant unit of program governance was the hierarchy defined by dispensary catchment area, ward, and district. Lack of congruence of randomization with bureaucratic context spuriously weakened prospects that the investigation of the CHW deployment hypothesis would succeed.

To implement the Connect trial, an interdisciplinary team was constituted, in which a systems learning exchange with Navrongo and Nkwanta implementers convened a participatory planning process to clarify requirements of adapting the Navrongo model to Tanzanian circumstances.^74^ CHWs were recruited from communities where they would be assigned, provided with a 6-month regimen of CHPS-like training, and then deployed to 49 randomly selected communities. A sample of 51 correspondingly randomized communities were selected where CHWs were not deployed.^75^ Legacy DSS data were marshalled for the decade prior to Connect permitting cause-specific mortality analyses^76^ and social determinants research that established preproject statistical balance of treatment and comparison conditions.^77^

For the initial 2 years, Connect operations proceeded as planned. A cadre of workers had been hired, trained for 6 months, paid a modest salary, and deployed to their home communities. Their primary health care regimen resembled the Matlab and Navrongo models. In year 3, however, problems ensued that were unrelated to project research hypotheses. Dispensaries served a catchment area typically composed of 3 or more communities. With communities specified as the primary unit of statistical randomization, Connect CHW deployment was misaligned with the operational design of the service system. Supervisors based in the dispensaries were responsible for both treatment and comparison communities. Moreover, district authorities were organizationally disconnected from the trial of CHWs, because both treatment and comparison conditions fell within the leadership domain. Managing experimental operational variance was contrary to managerial norms. So long as service operations were managed by the IHI research team, this structural anomaly did not detract from Connect implementation. But, in midstream, after 2 years of operation, the National Steering Committee requested the transfer of all frontline logistics operations from the IHI project team to local MOHSW authorities. The provision of essential supplies immediately broke down Connect because the national program logistics system was unprepared for the management of Connect CHW supply requirements. CHWs soon lacked essential supplies, causing their community credibility to evaporate and their impact on health and survival to atrophy in the final 2 project years.^77^ Although initial Connect results were impressive, the policy impact of the completed project was compromised by subsequent failure (Box 4). A planned phase 3 replication trial never emerged and systematic utilization of Connect for national scale-up was suboptimal. The premature embedding of Connect in a fragile health system undercut its utilization. Connect has contributed learning to the national program, but its operational design has not been scaled up.

BOX 4 **The Value of Strategic Isolation**

Connect was a test of the hypothesis that community health worker (CHW) deployment could save childhood lives. It was not a test of district Ministry of Health and Social Welfare (MOHSW) leadership capacity to manage a community program. The Connect protocol was an emerging success story that was disrupted by a premature shift of logistics management to a national MOHSW system that was experiencing serious organizational malaise. Embedding a district-level trial into this dysfunctional context disrupted the routine supply of essential medicines to CHWs, compromising their effectiveness and the statistical elegance of the Connect design. Without continuous supplies for essential services, CHWs lost credibility and Connect lost effectiveness in its final 2 years of operation. If Connect had functioned as an autonomous project, its final outcome would have been consistent with its successful first 2 years of operation. However, when final results were available, the project lost credibility. Replication research was never attempted. Impact on scale-up was marginal rather than central to program planning.

Although initial Connect results were impressive, the policy impact of the project was compromised by subsequent failure.

## DISCUSSION

**FIGURE 1:**
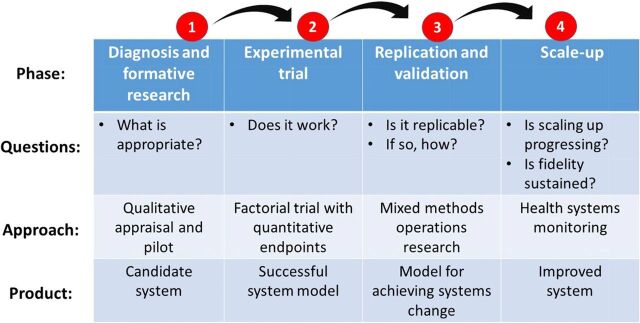
Research Phases Associated With Developing Community-Based Primary Health Care in Bangladesh and Ghana Adapted from Nyonator et al.^78^ and Awoonor-Williams et al.^79^

The 3 cases represent a common commitment to applying embedded research to a phased process for developing community-based primary health care systems (Figure 1).^78^ Despite common endpoints, theory, and embedded science methods, implementation outcomes differed because contrasting challenges were encountered, each requiring distinct strategies to address them. Fielding phase 1 investigations addressed the need for a program implementation strategy. Phase 2 experiments tested the operational model that emerged from phase 1. Phase 3, in Bangladesh and Ghana, transitioned research from testing impact to investigating the appropriate replication processes for achieving operational change. Phase 4 research monitored scale-up, either by supporting implementation processes or by indicating need for redirection and reform. Yet, all 3 examples encountered unresolved challenges.

Tables 1 to 3 cite attributes of implementation science design together with contradictions that each of the 3 embedded science initiatives encountered. Table 1 focuses on planning, Table 2 on operations, and Table 3 the utilization of results.

**TABLE 1.** Contradictions Associated With the Planning of Embedded Implementation Science and Case Study Examples of Strategies for Resolving Contradictions

Attribute	Core Strategies of Implementation Science	Strategic Adjustments of Embedded Science	Contradictions Associated With Strategic Adjustments	Case Study Resolution of Contradictions
Goal	Problems are identified *a priori* and resolved through researcher controlled hypothesis testing and dissemination.	Organizational change and development requires joint researcher and host agency goal setting.	Goals are defined in terms of endpoint hypotheses to be tested rather than host agency goals for testing means of achieving system change.	Retain, but subordinate, primary health and demographic impact research to implementation research as an integrated and continuous process.^4^
Outcome evaluation	Statistical inference is based on observation of treatment and counterfactual endpoints, with units of observation conforming to power requirements.	Improved host agency functionality and impact	Protocols define project start and end dates, endpoints, and hypotheses, whereas organizational change is a continuous, open-ended, and multi-faceted process.^10^	Phase in research as a process that fosters continuous utilization and action.^44^ Avoid ending learning processes just because a protocol has been completed.^2^
Leadership	Researchers in directive, independent, and autonomous roles with outreach to decision makers and managers at the end of investigation.	Collaboration of host agency and research partner leadership	Researchers assume directive, independent, and autonomous roles and episodically communicate health and demographic outcomes to host agency counterparts. Managers' roles are defined by bureaucratic and organizational norms.	Host agency managers representing each level of the investigative process are appropriately teamed with research counterparts at each system level.
Ownership	Host agency audience through “steering committees” and end of the project dissemination.	Subordination of research leadership to host agency governance. Joint dissemination.	Leadership malaise in the host agency can permeate an embedded research system, diluting rigor and compromising research implementation.^54^^,^^80^	Develop a partnership of research leadership with host agency institutional structures, but maintain an autonomous research operation.
Scientific rigor	Study designs conform to conventional criteria for statistical inference.	Studies embrace process research, mixed methods research designs, and multilevel analyses in concert with the norms of statistical inference.	Constructing the counterfactual is essential but inconsistent with management operations that span all organizational levels.^90^ Acquiring meaningful numbers of randomized organizational units for observation and statistical inference is impossible.^92^	Intervene with treatment and counterfactual conditions that conform to the host organizational structure. Use plausibility trials^56^^,^^85^ with statistical methods for non-experimental designs.^60^
System relevance	Systems thinking provides frameworks for data capture and analysis. Optimize costing for achieving clear and unequivocal results for hypothesis testing.	Systems thinking includes partnership arrangements and research activities that reflect units of the host agency organization. Establish researcher and host agency collaboration on operational costing and costing research.	Contexts where implementation science is needed most are settings where systems research is most challenging to conduct.^92^ Systems research requires multilevel longitudinal data that are complex to capture, manage, and analyze.^90^^,^^92^ Prospects for utilization are enhanced if costs are clear, but organizational change often incurs costs that are impossible to predict.	Utilize replication studies to disperse research in all relevant cultural and ecological contexts. Configure learning localities that are consistent with program organizational units at each level of the system.^44^ Restrict implementation financing to affordable and replicable activities. Prioritize costing analyses for replication and scale-up phases.^100^^,^^101^

**TABLE 2.** Contradictions Associated With the Process of Conducting Embedded Implementation Science and Case Study Examples of Strategies for Resolving Contradictions

Attribute	Core Strategies of Implementation Science	Strategic Adjustments of Embedded Science	Contradictions Encountered by Embedded Implementation Science	Implications for Resolving Contradictions
Teamwork	Constitute teams according to technical functions.	Delineate implementation and research teams.	Research teams and implementation teams have contrasting skills, orientations, and roles.	Configure at each level of the system “learning localities” where the pursuit of excellence is a collaborative endeavor that integrates implementation with investigation.
Simplicity	Develop measureable indicators of endpoints and possible confounders.	Focus on indicators that are commensurate with host organizational data capture, analysis, and communication capabilities.	Research and implementation integration is complex to undertake, but simplicity is often essential for fostering organizational change.^105^^,^^106^	Employ mixed methods research and knowledge management to promote understanding of essential processes and outcomes.^44^^,^^45^^,^^98^
Replicability	End of project terminates further research on replication or scale-up.	Design projects to facilitate subsequent replication and scale-up.	Developing learning systems requires focused inquiry in localities where interventions can be tractably managed. Managers often seek investigation that is immediately relevant to large-scale operations.^44^^,^^45^	Plan phases in advance^99^ that (i) diagnose systems requirements, (ii) test impact, (iii) test replication, and (iv) scale up based on replication lessons.
Fidelity	Fidelity of interventions to themes appearing in the scientific literature.	For longitudinal research on scaling up, develop communication mechanisms that ensure widespread host agency understanding of the evidence justifying change.	Primary science generates knowledge about impact without providing knowledge about change processes.^90^ Adapting to unanticipated changes is essential to scale-up.^100^ Fidelity to research outcomes is often incompatible with flexibility.	Develop “learning localities” for catalyzing the geographic spread of implementation. Integrate learning into national systems planning processes. Avoid advocacy focusing solely on “success” without also publicizing challenges and failure.^88^

**TABLE 3.** Contradictions Associated With Utilizing Embedded Implementation Science for Policy and Action

Attribute	Core Strategies of Implementation Science	Strategic Adjustments of Embedded Science	Contradictions Encountered by Embedded Implementation Science	Implications for Resolving Contradictions
Curation of knowledge	Publish results and disseminate findings to host agency and research audiences.	Develop knowledge-sharing mechanisms.	Science is disseminated by modes of communication that have limited currency among donors, decision makers, implementers, and managers.	Develop a multimethod knowledge management system for research advocacy,^105^ and build participatory learning and exchanges into research operations.^71^^,^^74^
Sustainability	Recommend utilization of research findings in the course of end-of-project dissemination activities.	Collaboration of researchers and host agency counterparts on research utilization strategic planning.	Planning research utilization is challenged by the institutionalization of dysfunction. Failure is therefore more sustainable than improvement. Research results may contradict existing organizational norms and policies.	Utilize research phase 3 replication research to investigate the determinants of sustainability.

### Contradictions Associated With Embedded Implementation Research Planning

Planning implementation research in collaboration with a host organization is a fundamental principle of embedded science. Yet, the process of collaborative planning is challenged by contrasting goals of researchers and implementers and alternative perspectives on process. Rows of Table 1 summarize challenges to embedded research planning. The goal of joint leadership for establishing host agency ownership is challenged by contrasting professional leadership norms of researchers and managers. Moreover, differing criteria for defining scientific rigor can be associated with contrasting perspectives on the relevance of results.

Planning implementation research in collaboration with a host organization is a fundamental principle of embedded science.

#### Precisely Defined and Measurable Goals

In each case, research achieved demographic and health impact for each phase in the research process. While this contributed to the credibility of each initiative, primary goals, as defined by host agency leaders, were more focused on demonstrating the feasibility of changing operations, the practicality of intervention components, and the operational integrity and quality of community health services. This focus on achieving systems change contrasts with conventional project-based fixed-duration health research whereby designs, protocol execution, and dissemination of outcomes are preplanned and governed by protocol. Amplifying this contradiction is the tendency for project completion to terminate further thinking about organizational change implications. Utilization of results is a research afterthought rather than an explicit objective worthy of investigation. Operational change may be fortuitous under such circumstances, but more typically, large-scale change does not occur or is not included as a topic of explicit research focus. Fully embedded science connotes a process of continuous institutionalized learning and action that never ends, just as effective management is an interminable goal.

Overarching research goals to change organizational structure or functioning differ from goals that concern biomedical experimental outcomes. In Bangladesh, organizational change was conducted in phases that were informed by continuous learning that was integrated into leadership activities and planning processes. Activities involved in setting up the Maternal and Child Health–Family Planning Extension Project were documented in ways that provided operational plans for the World Bank's lending agreement. Rolling out the extension project simultaneously rolled out the World Bank's plan well before project demographic and health outcomes were known. This process of integrated knowledge management and curation was also characteristic of the Ghana case,^80^ where the process of launching Navrongo and Nkwanta operations led to the 1999 CHPS policy. Navrongo completed its protocol in 2003, and Nkwanta replication activities continued until 2004. Yet national CHPS scale-up commenced in 2000. This anomalous timing of action was done for a reason: The focus in each case was achieving systems change rather than testing hypotheses on health and demographic outcomes. If phase 3 results had showed that replication failed to achieve phase 2 results, a new round of phase 1 diagnostic learning would have commenced with the goal of developing phase 4 reform.

Overarching research goals to change organizational structure or functioning differ from goals that concern biomedical experimental outcomes.

#### Decision-Making Processes

A manager assessing the impact of implementation research is focused on improving operations rather than the health or demographic outcomes that are external to direct management control. The integration of research operations with host agency organizational structure contributes directly to research credibility. Procedural monitoring with multi-method research is critical to the conduct of embedded science. Contradictions arise, however. Temporal congruence can be inconsistent with the continuous provision of information for program planning and decision making.^81^ Research timelines may encounter delays or adjustments in order to align technical activities with counterpart planning cycles. This adjustment process can cause projects to overrun timelines and deplete resources before final analyses or dissemination activities can be conducted. Moreover, abandoning temporal congruence to ensure compliance with protocol timelines can detract from official interest in research operations.

In Bangladesh, this problem was addressed by integrating project information generation cycles into the national planning cycle, so that project communication could provide direct input into the World Bank lending agreement. But, in Ghana and Tanzania, temporal integration of research-based learning cycles with the timing of national planning and donor agreement cycles was never possible. In Ghana and Bangladesh, health authorities addressed this problem by integrating project reporting into routine communication mechanisms. In Ghana, however, delays were associated with temporal discordance of mechanisms that provided resources for implementation research with timing cycles that funded CHPS implementation.

#### Host Agency Ownership

Establishing host agency ownership involves collaboration in the process of research management, project implementation, and dissemination.^82^^–^^84^ In each of the 3 case examples, integration of scientific leadership into planning units of the host government was facilitated by host agency research skills that were commensurate with leading protocols, conducting investigations, and interpreting results for national program utilization. Despite this potential for joint ideational development, leadership sharing is alien to most health bureaucracies, which require leadership to be singular, directive, and decisive. However, external research support often involves external technical advisors who depend upon funding, professional recognition, and achievement criteria. A totally embedded academic or research agency partner will have career goals that are at odds with ceding ownership and ideational leadership to program implementers. Indeed, even the scholars who promote embedded science only rarely practice embedded science in the course of their work. Some degree of strategic isolation of research is appropriate. Independent ideational leadership is critically important, totally justified, and essential to the advancement of implementation science. Such capabilities are often unavailable in the institution that implementation research has targeted for change. Researchers are often obligated to defy embedded ownership goals and take charge of some operations that research is testing, as in the Matlab and Ifakara examples.

Despite the potential for joint ideational development, leadership sharing is alien to most health bureaucracies, which require leadership to be singular, directive, and decisive.

Disengagement of researchers with host agency institutions can be particularly critical to developing innovations during the initial phases of implementation research. In many instances, research associated with discovery and organizational diagnosis would not survive if bureaucratic scrutiny was embedded with research operations. This differentiation of roles also applies to host agency leaders. Administrative issues that they, as owners, recognize as meriting investigation may be of little perceived importance to researchers. While the pursuit of joint ownership may be critical to embedded science, not all phases in the development process benefit from joint leadership.

Embedded science ownership assumes that all essential partners are included in the vital system of decision making. If donors are positioned to shape policy, action, and the research itself, they must be embedded in the systems development process, just as the World Bank was a systems investor in Bangladesh. But, if donor agreements that fund research or implementation impose mechanisms that extract ownership, leadership, or ideational integrity from embedded processes, no amount of embeddedness among other players will matter.

#### Statistical Rigor

The pursuit of statistical rigor can confound embedded project designs. For example, Ghana's GEHIP project failed to have an impact the survival of children aged 1–4 years because the mortality decline in comparison areas was equivalent to trends in treatment areas. This pronounced improvement in child health was the outcome of successful strengthening of the Integrated Management of Childhood Illness care system throughout the UER. In the embedded science paradigm, the Regional Director of Health Service was a co-investigator, whose successful and fully appropriate leadership led to health systems improvements in comparison districts that spuriously introduced statistical failure when comparison area trends were found to match progress in treatment areas.^56^^,^^58^

Connect in Tanzania also illustrates challenges. Randomization of community units of observation complied with the criteria of rigorous randomized cluster trials. Yet, this feature of the design was its major shortcoming. Achieving organizational congruence for decision making that is evidence based is more critical to embedded science than optimizing observational congruence with criteria for statistical decision making.^85^

#### System Relevance

The institutional context of trials influences the credibility of results.^86^ Health systems research is often implemented as a project that is optimized for achieving endpoint success. Resources, site location selection, staffing, and organizational arrangements are configured in ways that ensure that objectives are achieved^87^ since scientific credibility and donor support usually require evidence of endpoint success. Failure as a theme in the health systems science literature is rare.^88^ This emphasis on achieving endpoint success can be particularly dysfunctional in Africa where contextual variance in organizational, cultural, linguistic, and ecological circumstances can be pronounced. In any country setting, findings emerging from a single location and provided with unusual features and resources can be dismissed as being irrelevant to national policy.^86^^,^^87^ In response to this challenge in Bangladesh and Ghana, interregional dispersion of embedded replication projects ensured that geographic context would not overshadow the programmatic relevance of results.^60^

Health systems research is often implemented as a project that is optimized for achieving endpoint success.

The relevance of embedded science is also enhanced if units of inquiry span levels of the host agency system. Although systems thinking gained currency with the dissemination of the World Health Organization (2007) systems strengthening framework^89^^,^^90^ apart from a few examples, systems strengthening trials remain rare in Asia and Africa^37^^,^^73^^,^^91^ because of the high cost, complexity, and organizational challenges of systems research.^92^

In all 3 cases, investment in innovation incurred incremental costs. Establishing cost compatibility with host agency budgets and generating host agency knowledge of these costs was important to deliberations on the utilization of results.^93^^–^^95^ Once operational impact had been demonstrated, prospects for utilization were enhanced. Contradictions arose, however, when budgeting and financial procedures of the host agency contrasted with the financial procedures associated with embedded research projects. Costs of innovation were affordable and yet procedurally awkward to undertake. However, in Bangladesh, costing information facilitated financing by the World Bank and offset the political risk of adding the FWA cadre to the system. But, in Ghana, the start-up costs of CHPS implementation was not the focus of external investment. Reforms instituted in 2009 specified budget lines that permitted district directors to plan the costing of health posts, equipment, and supplies. Congruence between research recommendations and capacity to act was enhanced. Politically embedded CHPS catchment areas enabled district directors to align revenue negotiations with domains of responsibility of local development officials. In Tanzania, costs were financed externally, but resources were managed by local government mechanisms. Connect was not only affordable, but its financial requirements were manageable within the host organizational system.^95^

### Contradictions Associated With Conducting Embedded Implementation Research

Conducting research and operational leadership as a partnership represents a fundamental principle of embedded science. Dual leadership is fundamentally challenging in any formal organization. In particular, conducting joint embedded science operations is challenged by the complications associated with integrating teamwork, by the challenge of simplifying investigation of problems that are complex, and by maintaining flexibility when sustaining fidelity is vital to maintaining compliance with evidence. Table 2 summarizes these contrasts and corresponding case study outcomes.

Conducting joint embedded science operations is challenged by complications from integrating teamwork, investigating problems that are complex, and maintaining flexibility.

#### Teamwork

As the 3 cases have demonstrated, the delineation of research from implementation functions can structure task autonomy rather than foster embedded collaborative task implementation. This dysfunction can be offset with multi-method interdisciplinary teamwork involving collaborative extraction of lessons spanning each level of the system,^94^^,^^95^ thereby facilitating investigation of hierarchy, structure, and function and fostering host agency stakeholder consensus that results are relevant to the system at large.^96^^,^^97^ But, total research integration is tantamount to contamination because managers are both the subject of research and the purveyors of implementation insights. Moreover, managing operations can be inconsistent with ceding authority to researchers, even if trials are testing ways to improve program performance. Partnership is possible if leadership focuses on mechanisms for achieving interdisciplinary teamwork, but more commonly, functional diversity constrains embedded management and research thinking.

#### Simplicity

Evidence-based change is an application of complexity science.^98^ Phases, milestones, and implementation processes are complex to understand, document, and explain. Complexity was addressed in Bangladesh by embedding research outcomes into routine government orders, plans, training guidelines, and internal memoranda. In Ghana, simplicity was achieved when field demonstration was combined with catalytic financing that enabled participants to pilot the system they were learning how to manage. Mixed methods research was used in all 3 case examples to integrate implementation process knowledge into managerial utilization of outcomes.^44^^,^^45^ However, this integration process is often too complex for donors and senior officials to embrace unless political action drives the integration process. Organized units were created, in each case, to create a bridge between research project teams and planning units. In Ghana, for example, a national order rearranged CHPS catchment areas to coincide with grassroots electoral areas, thereby aligning popular support for CHPS with District Assembly member electoral aspirations. This simplified district health leadership actions required building political support for defraying CHPS start-up costs with district development revenue. And, to achieve organizational congruence, the national office responsible for health planning, known as GHS Policy Planning Monitoring and Evaluation Division, was charged with the task of monitoring all CHPS-related research in Ghana. In Bangladesh, a World Bank–financed management development unit was convened to support the communication of project results to planners. In Tanzania, a planning unit of the MOHSW was charged with the task of monitoring project progress.

#### Flexibility and Replicability

Scalability is often an afterthought rather than a process that is antecedent to research.^99^ This was the principal limitation of the Matlab phase 2 FPHSP that the phase 3 extension project was convened to address. Phase 3 was crucial to catalyzing utilization of Phase 2 outcomes. Program planners at the World Bank had assembled the initial draft of Population and Health Plan Three in Washington without review of extension project results. This procedural isolation was perceived by the World Bank's officials to be essential to organizing lending, because World Bank procedures were complex, requiring careful compliance with preparation timelines. Co-financing arrangements that added critically needed resources also added complexity. Nearly 60% of the $243 million agreement was foreign aid contributed to the lending agreement by European governments and Canada. Procedural momentum for configuring a large and complex agreement required fidelity to prearranged timelines rather than fidelity to research findings. As a result, the text of the initial agreement was voluminous, yet imported from afar, and tangential to health development needs.

Scalability is often an afterthought rather than a process that is antecedent to research.

In response to the World Bank's procedural agenda, the Bangladesh MOHSW deliberations focused on sustaining the program and complying with the World Bank's leadership with extension project lessons initially assuming the character of a disruption of the process of developing the vitally important World Bank agreement. But, the products of extension project implementation soon provided the Bangladesh government with content to use in negotiating terms specified in the World Bank's agreement. By providing the Bangladesh government with documented evidence that supported its procedural ownership of the borrowing process, the extension project catalyzed national program adoption of the Matlab service model well before the extension project demographic results were attained.

In Ghana, general policy and programmatic decisions to scale up results were taken prior to the launching of Navrongo research. However, the full range of considerations for financing scale-up lacked the strategic integration of research outcomes with donor processes that had supported scale-up in Bangladesh. In fact, throughout the CHPS development process, donors were only peripherally embedded in the initial planning process. Based on donor understanding of the potential impact of CHPS, but insufficiently engaged with implementation processes, donors engaged contracting mechanisms for supporting CHPS that extracted ownership from GHS-embedded partnership arrangements. Scaling down operations to tractable levels of district managerial learning had catalyzed scaling up at the district level,^60^ where effective scaling-up progressed if district managers developed 1 or 2 learning communities. By utilizing their functional CHPS operations as demonstration zones, leadership action could spread CHPS implementation.^53^ Lacking mechanisms for centralizing this fundamentally decentralized process, embedded science failed to foster appropriate donor investment.

Scaling-up in each district was confronted with a fundamental contradiction represented by the requirement of flexibility and fidelity. Utilization of research implies fidelity to the evidence, while managerial flexibility is equivalently essential to the process of achieving change. Flexibility to permit district leadership to progress is essential to CHPS development. However, maintaining balance between fidelity and flexibility requires ensuring an element of research autonomy that is somewhat at odds with the tenets of embedded science.

Utilization of research implies fidelity to the evidence, while managerial flexibility is equivalently essential to the process of achieving change.

In all 3 case examples, project steering committees were constituted to ensure that activities were both compliant with protocols and adaptable to changing operational needs. To protect fidelity, mechanisms insulated research teams from bureaucratic constraints; yet, corresponding mandates permitted managers to engage in change that implementation research required. In Bangladesh, the institutional autonomy of the icddr,b insulated extension project research from bureaucratic constraints and district managers in extension project areas were permitted to alter training, supervision, and worker deployment schemes. Similarly, in Tanzania, the IHI field research stations were essentially autonomous from MOHSW oversight and managers were provided with mandates to support CHW deployment and supervision. In Ghana, research units of the GHS are separated from directorates responsible for implementation, but when implementation research is undertaken, the existing GHS management system retains control of operations. In all 3 settings, research projects had autonomous accounts, technical research staffing, and logistics capabilities that permitted operational independence and flexibility of research operations that regional and district management teams lacked. Yet, care was taken to ensure communication, reporting, and accountability to the organizational hierarchy of the host agency system.

### Contradictions Constraining the Institutionalization of Results

Research establishing that implementation can improve health or well-being contributes little to understanding how utilization of such findings can proceed. This contradiction was addressed by the cases, each requiring the pursuit of implementation flexibility in conjunction with fidelity to research lessons. Knowledge was not just created by research; learning was institutionalized. Initiatives evolved as continuous and iterative learning capabilities rather than projects that ended, with their termination also ending knowledge curation (Table 3).

#### Curation of Knowledge

Mechanisms for scientific dissemination are typically designed to communicate with other researchers rather than to optimize evidence-driven decision making.^100^ Efforts to address this problem were pursued in each case (Table 3). In Bangladesh, extension project communication was integrated into MOHSW circulars. In Ghana, international responsibility for the dissemination of science was not only shared with policy makers, but was also generated as a participatory process involving policy makers who had responsibility for using this knowledge for program development. In Tanzania, cell phone technology was used to foster knowledge sharing among frontline workers. In each example, community stakeholders contributed material to a series of documents, news articles, web traffic, site visits, and other forms of awareness building for institutionalizing knowledge emerging from research operations.^7^

Mechanisms for scientific dissemination are typically designed to communicate with other researchers rather than to optimize evidence-driven decision making.

#### Sustainability

The pursuit of sustainability is an important goal of embedded implementation science. Yet, researching sustainability is typically impossible within the timeframe of research projects. Since failure is more sustainable than success, sustainability per se should be researched in the final phase of embedded implementation research. In each case, phase 3 permitted costing studies, staff responses, and other practical considerations such as implementation milestone clarification.

A process of continuous temporal congruence of research with program planning is critical to ensuring embedded science decision making.^84^ Researchers may be required to adjust plans and activities to be commensurate with counterpart planning and activity cycles. But such an adjustment process can overrun timelines and divert resources from final analyses or dissemination activities. If temporal and task congruence is ignored, official interest in research operations can wane before results are available.

In Bangladesh, this problem was addressed by integrating project information generation into the national planning cycle, so that the Third Population and Health lending agreement was supported by output from project activities. But, in Ghana and Tanzania, temporal integration of research-based learning cycles with the timing of national planning and donor agreement cycles was never possible. In Ghana, the GHS attempted to address this problem by integrating project reporting into routine communication mechanisms. Temporal discordance of mechanisms that provide resources for research represents a challenge for embedded science. Timing of evidence production can be inconsistent with cycles associated with the utilization of results for organizational change.

### A Synthesis: Agile Science

In 2001, a computer engineering working group convened a review of innovations that could improve software development compliance with user needs. Together they constructed an “Agile Manifesto” that has had a transformative impact on best practices in software engineering and has influenced innovations in agile science.^101^ Table 4 illustrates how an agile science perspective can contribute to resolving methodological challenges and contradictions encountered by the case studies. The table aligns the key attributes of embedded implementation science with the 12 principles of agile development to suggest ways in which the contradictions identified in the case studies were or can be resolved.

**TABLE 4.** Implications of Lessons From the Principles of Agile Science and Case Example for an Agile Paradigm for Embedded Implementation Research

Attribute	The Agile Working Group's 12 Principles of Agile Science^101^^,^^a^	Agile Embedded Science Implications
Goal	“Our highest priority is to satisfy the customer through early and continuous delivery of valuable software [program improvements].”	Problem identification is a continuous process. Owing to contextual complexity and uncertainty, problem details and solutions cannot always be identified in advance.
Outcome evaluation	“Continuous attention to technical excellence and good design enhances agility.”	Monitor compliance with implementation goals continuously with evaluation criteria that continuously shift, as needed. Subordinate demographic and health hypothesis testing to implementation process evaluation.
Leadership	“The best architectures, requirements, and designs [research strategies] emerge from self-organizing teams.”	Problem identification and candidate solutions can be defined by anyone in the research or host agency teams. Peer leadership is encouraged. Project leadership is systemic and multileveled, and it is the outcome of collaborative investigation of appropriate system development needs.
Ownership	“Business people [Host agency participants] and developers must work together daily throughout the project.”	Establish host agency and research joint ownership. Participatory decision making throughout the process of organizational development.
Scientific rigor	[Not relevant]	Develop credible results that focus on implementation processes and outcomes.
System relevance	Working software is the primary measure of progress “Deliver working software [or research products] frequently, from a couple of weeks to a couple of months, with a preference to the shorter timescale.”	Achieve concordance of research operations with host agency structure and functions. Assess costs and design research to demonstrate affordability. Open-ended, iterative, and continuous sharing of information and review of progress. Timing of phases governed by host agency planning and decision processes.
Teamwork	“At regular intervals, the team reflects on how to become more effective, then tunes and adjusts its behavior [or strategies] accordingly.”	At regular intervals, program managers review feedback to implementers and researchers to detect departures from quality or the need to adjust research or implementation strategy. Roles are integrated for research and host agency counterparts by implementation function.
	“Build projects around motivated individuals. Give them the environment and support they need, and trust them to get the job done.”	Build teams around champions who are successful communicators of innovation. Foster peer leadership through exchanges.
Simplicity	“Simplicity—the art of maximizing the amount of work not done—is essential.”	Simple solutions are preferred over more complex interventions. Complexity determined by host agency targeted changes to be investigated.
Replicability	“Welcome changing requirements, even late in development.”	Intervention targets, processes for monitoring, and evaluation procedures can be changed by evolving host agency priorities.
Fidelity	[Not relevant]	Intervention targets, processes for monitoring, and evaluation procedures can be changed by evolving host agency priorities.
Curation of knowledge	“The most efficient and effective method of conveying information to and within a [software] development team is face-to-face conversation.”	Direct communication between host agency and research team is essential. Integrate the process of generating evidence and outcomes with the process of utilizing evidence for decision making.
Sustainability	“Agile processes promote sustainable [software] development. The sponsors, developers, and users should be able to maintain a constant pace indefinitely.”	Research activities and processes are pursued at a pace that can be maintained indefinitely. Outcomes are delivered continuously as a regular part of research operations. Investigation is embedded in change processes that are continuous and never ending.

Adapted from similar tables by Nerur et al.^107^ and by Flood et al.^109^

The agile principles attest to ways of improving embedded science, beyond the strategies that the 3 case studies provide. The overarching goal of applying agile science to health systems development is summarized by Kessler and Glasgow.^102^

*Randomized controlled efficacy trials using precisely defined interventions and highly selected participants … when applied to the other major issues facing health care today … are limited in their ability to address the complex populations and problems we face. … Pragmatic, transparent, contextual, and multilevel designs that include replication, rapid learning systems and networks, mixed methods, and simulation and economic analyses to produce actionable, generalizable findings that can be implemented in real-world settings is suggested. This shift would include greater focus on the needs of practitioners, patients, payers, and policymakers and generate more relevant evidence.*

Five themes of agile software engineering could contribute to improving the application of embedded implementation science to health system strengthening.^103^ First, the goal of focusing on implementation objectives would be enhanced by methodologies that include the estimation of scales and composite indices of service readiness, leadership acumen, or other indicators of implementation functionality.^104^ Second, agile leadership respects the importance of peer leadership and recognizes that strong leadership is an outcome of the successful efforts of capable and creative subordinates. Third, continuous team evaluation and review is emphasized in the agile paradigm. Fourth, establishing functioning systems that can be continuously improved is of greater importance than awaiting achievement of optimum endpoints. Fifth, timing is critical. In the linear approach shown in Figure 1, each phase can be needlessly prolonged if protocol completion governs the duration of phases.

The coterminous implications of case examples with the Table 4 tenets of agile science invites consideration of an amalgamation of strategies and implications. An overarching research design could replace the piecemeal approach of the case studies, build upon engineering agility ideas, and obviate the tendency of some implementation researchers to reject embedded science altogether. Agile and embedded scientists alike share a recognition of the importance of employing phased stages in system development.^105^^,^^106^

**FIGURE 2:**
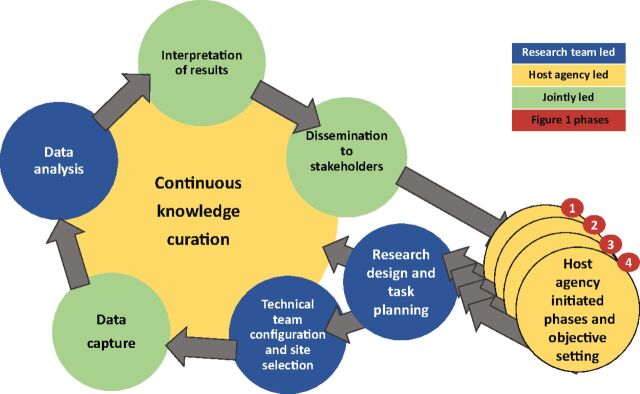
The Agile Science Process of Health Systems Strengthening

Figure 2 highlights elements of the process implied by such an amalgamation. It portrays the iterative application of agile principles for inference, decision making, and action. These agile cycles commence with the host agency identifying objectives for change and improvement. Launching deliberations can be informed by previous research activities, tacit system knowledge, the scientific literature, or advisory support from research partners. The agile research team then proceeds to design studies that refine the design of interventions, test the effectiveness of interventions, and clarify requirements of achieving organizational change that address host agency objectives for large-scale implementation. Dissemination is a continuous component of the process, with options for taking many alternative mechanisms based on the needs of the host agency.

Agile and embedded scientists alike share a recognition of the importance of employing phased stages in system development.

Figure 2 can serve as a supplement to the linear phased processes shown in Figure 1. Each phase can have multiple iterations of discovery, investigation, and results generation until the objectives of the phase are satisfied. As in Figure 1, formative research is associated with phase 1. Phase 2 corresponds to trial, experimentation, or quantitative appraisal of prospects that formative knowledge gained in phase 1 could actually work, as intended. Phases 3 and 4 have corresponding agile cycles, with knowledge curation as the outcome of each recurrent cycle. But irrespective of phases, the Figure 2 cycle applies within each phase. This iteration, with larger phases is analogous to the Agile Unified paradigm, a software development process that has phases of product conceptualization, elaboration and trial, refinement, and deployment.^107^

Taken as a system of work, the Figure 2 cycle of ideational development, evaluation, and change could form the basis for embedded implementation science.^26^^,^^106^^–^^110^ Doing so would optimize the pursuit of embedded science goals while sustaining the overarching need for rigorous science.

The centrality of knowledge curation in Figure 2 portrays the institutionalization of system learning. In the agile paradigm, the combination of documenting learning, responding to learning, and disseminating learning to relevant stakeholders converts knowledge management processes into interactive and institutionalized system learning. If all stakeholders are aware of curated knowledge and informed by each round of problem solving, then knowledge curation is possible. By maintaining agile team field investigators who have a mandate to engage in regular dialogues with host agency counterparts, each cycle diagnoses problems, tests solutions, and advises the agile team leaders on appropriate responses to emerging lessons. Whether the process represents an agile acquisition of discrete decision-making guidance or a multi-year process of institutional reform is determined by the user in concert with the agile research generated evidence.

As Table 4 suggests, agile investigation and action can serve multiple purposes that include the introduction of new organizational strategies or processes that are focused on organizational reform. Agile embedded implementation science is not a project; rather, it is a process of evidence-driven organization development. By sustaining the agile process, knowledge curation and continuous action are recurrent, securing systemic organizational learning about implementation challenges and successes.

Agile embedded implementation science is not a project; rather, it is a process of evidence-driven organization development.

## CONCLUSION

The embedded science that guided community-based primary health care development in Bangladesh, Ghana, and Tanzania attests to the value of subordinating the generation of research results to researching the process of changing the way that programs work. Doing so requires a system of action and decision making that integrates the tools of science into the process of managerial planning and decision making.^14^^,^^15^^,^^100^^,^^105^ Although the pursuit of this goal will encounter intrinsic contradictions, strategies that case examples pursued piecemeal could be assembled into a combined approach that would strengthen the application of embedded science. Agile science has functioned well to that end for organizations engaged in developing software, medical technologies, and engineering innovations. As our case examples illustrate, the elements of agile science are also feasible in settings that are not yet equipped with advanced technologies. Assembling these elements as an agile extension of embedded research could sustain scientific rigor while optimizing the prospects for results to be put to use.

## Notes

**Peer Reviewed**

**First published online:** February 9, 2021.
